# A Crohn's Disease Patient Found to Have *Entamoeba histolytica* Infection Causing Pyogenic Liver Abscess

**DOI:** 10.1155/2023/9936613

**Published:** 2023-07-31

**Authors:** Hannah Saven, Ashton Harmacinski, Andrew Canakis, Uni Wong

**Affiliations:** ^1^University of Maryland Medical Center, Baltimore, MD, USA; ^2^Division of Gastroenterology and Hepatology, University of Maryland School of Medicine, Baltimore, MD, USA

## Abstract

*Entamoeba histolytica *is a parasite that typically causes amoebic dysentery but can result in complications such as pyogenic liver abscess. Patients with inflammatory bowel disease often take immunosuppressive therapies that make them more susceptible to such infections. Notably, parasitic infections in this context are rare in nonendemic areas. We describe a 57-year-old man who recently started infliximab therapy for Crohn's disease and presented with fever and right upper quadrant pain. While hospitalized, this patient was diagnosed with *Entamoeba histolytica* liver abscess. This case demonstrates that parasitic infections should be considered early in immunocompromised patients with inflammatory bowel disease.

## 1. Introduction

Anti-tumor necrosis factor (TNF) agents are the mainstay of therapy for moderate-to-severe Crohn's disease (CD). While effective in preventing the complications of inflammatory bowel disease (IBD), anti-TNF has been associated with an increased risk of infection. Although bacterial and viral infections have been previously reported [1], parasitic infections are uncommon [2]. In this report, we describe a patient with CD who developed *Entamoeba histolytica* liver abscess while receiving anti-TNF therapy.

## 2. Case Presentation

A 57-year-old male with a history of inflammatory colonic Crohn's disease diagnosed in 2017 underwent colonoscopy in September 2021 for disease assessment, which revealed endoscopically active colitis with a shallow ulcer in the rectum and a large circumferential patch of ulcerations in the right colon. The patient was then started on infliximab, an anti-TNF agent. After two induction doses of infliximab, the patient presented to the emergency department with cyclic fevers (101–104°F) for ten days. The patient experienced cramping right upper quadrant pain, night sweats, headaches, and shaking chills every evening with resolution of all symptoms during the day. He denied having any ill contacts or recent international travel. He had a history of international travel to Kuwait, Qatar, Egypt, Japan, and Italy in the early 2000s.

**Figure 1:**
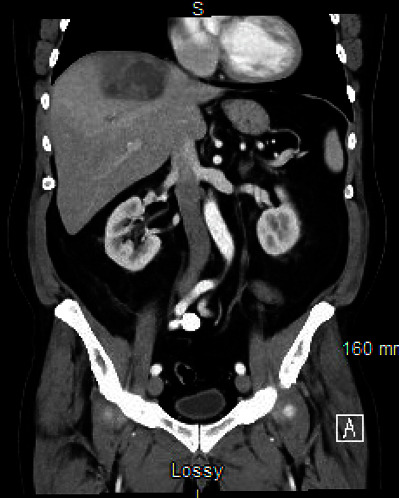
Two hypoenhancing lesions within the right hepatic dome (9.2 cm and 3.5 cm) compatible with hepatic pyogenic abscesses.

**Figure 2:**
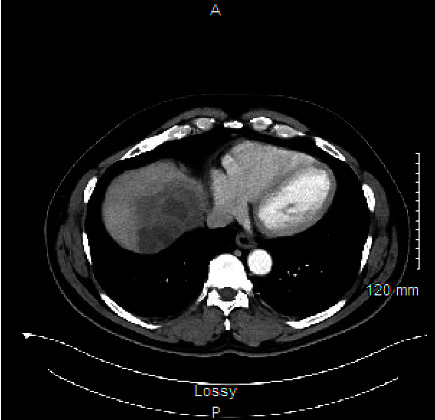
Axial view of hepatic pyogenic abscesses.

On examination, the patient was febrile (up to 101.8°F), mildly tachycardic (94 beats per minute), and on room air. Abdominal examination revealed mild tenderness on palpation of the right upper quadrant without rebound or guarding. Laboratory work was notable for leukocytosis (12.8 × 103/*μ*L) with a mild elevation in neutrophil count (9.2 × 103/*μ*L). Other pertinent labs are as follows: aspartate aminotransferase 50 IU/L, alanine aminotransferase 49 IU/L, alkaline phosphatase 71 IU/L, total bilirubin 0.5 mg/dL, prothrombin time 18.2 seconds, international normalized ratio 1.75, and activated partial thromboplastin time 33.4 seconds. Computed tomography (CT) of the abdomen and pelvis revealed two hypoenhancing lesions in the liver measuring 9.2 cm and 3.5 cm consistent with pyogenic liver abscesses (PLA) (Figures 1 and 2).

Chest radiography, urinalysis, and blood cultures results were unremarkable. *Echinococcus* and *Entamoeba histolytica* antigen tests were performed. CT-guided percutaneous abscess drainage with pigtail drain placement was also performed. Gram staining and culture of the liver abscess aspirate were negative. The patient was empirically treated with piperacillin/tazobactam for Gram-negative coverage of a suspected intraabdominal infection. On day seven of hospitalization, the *Entamoeba histolytica* enzyme immunoassay returned positive results. The patient was then treated with metronidazole for two weeks followed by one week of paromomycin therapy to eliminate any remaining intraluminal cysts. After one week of metronidazole therapy, stool antigen testing was negative for infection. The patient was subsequently examined in the clinic in January 2022 and had complete resolution of all symptoms while he was off infliximab treatment. In June 2022, the patient underwent a follow-up colonoscopy and was negative for endoscopically active disease. He will undergo a surveillance colonoscopy to monitor the recurrence of Crohn's disease.

## 3. Discussion

Pyogenic liver abscess is a rare manifestation of extracolonic inflammatory bowel disease. One large cohort study in Taiwan, where the incidence of EH is low, suggested an increased incidence of PLA among IBD patients, 6.72/10,000 person-years compared to 4.06/10,000 person-years in patients without IBD [3]. In healthy patients, EH often colonizes the colonic mucosa without causing any symptoms. The friable, damaged colonic mucosa exhibited in many IBD patients allows for easy invasion of these microbes and their translocation to the liver via the portal vein [4]. Long-term steroid treatment and immunosuppressive medications which are the mainstay of IBD treatment may also predispose patients to developing PLA of other infectious etiologies, such as *Klebsiella pneumoniae* and enteric bacteria such as streptococci and *E. coli*, which are the most common pathogens in patients living in the United States [4, 5]. Therefore, a wide variety of microbes should be considered pathogens in the PLA of patients with IBD.

PLAs caused by EH are rare outside endemic areas, such as India, Africa, Mexico, and Central and South America. When it occurs in nonendemic areas, it is typically diagnosed in individuals who partake in oral-anal sexual contact [6]. This patient did not report recent international travel or oral-anal sexual contact prior to diagnosis but did have a significant remote travel history to endemic areas. We hypothesized that this patient may have been colonized with EH from remote international travel that became invasive once the patient was immunosuppressed. Additionally, it may be difficult to discern diarrhea in IBD flares from invasive EH infection, which in turn delays diagnosis [4]. This was the case for our patient, who initially attributed his symptoms to active CD and only sought medical attention when unusual symptoms of fever, chills, and right upper quadrant pain developed. Therefore, EH infection should be considered early in immunosuppressed patients presenting with PLA.

## Data Availability

No underlying data were collected or produced in this study.
